# Protective effect of palm vitamin E and α-tocopherol against gastric lesions induced by water immersion restraint stress in Sprague-Dawley rats

**DOI:** 10.4103/0253-7613.41042

**Published:** 2008

**Authors:** Ibrahim Abdel Aziz Ibrahim, Kamisah Yusof, Nafeeza Mohd Ismail, Nur Azlina Mohd Fahami

**Affiliations:** Department of Pharmacology, Faculty of Medicine, Universiti Kebangsaan Malaysia; Kuala Lumpur, Malaysia; 1 Department of Pharmacology, Faculty of Medicine, Universiti Teknologi MARA, Shah Alam, Salangor, Malaysia

**Keywords:** Gastric lesions, gastric PGE_2_, palm vitamin E, water immersion restraint stress, α-tocopherol

## Abstract

**Objective:**

Stress can lead to various changes in the gastrointestinal tract of rats. The present study was designed to compare the effect of palm vitamin E (PVE) and α-tocopherol (α-TF) supplementations on the gastric parameters important in maintaining gastric mucosal integrity in rats exposed to water immersion restraint stress (WRS). These parameters include gastric acidity, plasma gastrin level, gastric prostaglandin E_2_ (PGE_2_), and gastric lesions.

**Materials and Methods:**

Sixty male Sprague-Dawley rats (200-250 g) were divided into three equal groups: a control group, which received a normal rat diet (RC), and two treatment groups, receiving oral supplementation of either PVE or α-TF at 60 mg/kg body weight for 28 days. Each group was further divided into two groups: the nonstress and stress groups. The stress groups were subjected to 3.5 h of WRS once at the end of the treatment period. Blood samples were then taken to measure the gastrin level, after which the rats were killed. Gastric juice was collected for measurement of gastric acidity and gastric tissue was taken for measurement of gastric mucosal lesions and PGE_2_.

**Results:**

Exposure to stress resulted in the production of gastric lesions. PVE and α-TF lowered the lesion indices as compared to the stress control group. Stress reduced gastric acidity but pretreatment with PVE and α-TF prevented this reduction. The gastrin levels in the stress group were lower as compared to that in the nonstress control. However, following treatment with PVE and α-TF, gastrin levels increased and approached the normal level. There was also a significant reduction in the gastric PGE_2_ content with stress exposure, but this reduction was blocked with treatment with both PVE and α-TF.

**Conclusion:**

In conclusion, WRS leads to a reduction in the gastric acidity, gastrin level, and gastric PGE_2_ level and there is increased formation of gastric lesions. Supplementation with either PVE or α-TF reduces the formation of gastric lesions, possibly by blocking the changes in the gastric acidity, gastrin, and gastric PGE_2_ induced by stress. No significant difference between PVE and α-TF was observed.

Stress is a condition that can affect psychological and physiological balances and lead to various pathological changes, including the formation of gastric ulcers.[1] The pathological basis for the development of these lesions has been postulated to be multifactorial and includes changes in the gastric acid secretion, disruption of gastric mucosal barrier, reduction of gastric mucosal blood flow,[2] inhibition of gastric mucus and bicarbonate secretion,[3] and inhibition of gastric mucosal prostaglandin synthesis.[4]

Stress causes the elevation of corticosteroids, which leads to vasoconstriction due to an increase in catecholamine.[5] The elevation of these hormones can also disrupt gastric motility through the increase in stomach contractions.[6] Vasoconstriction and gastric hypermotility can impair gastric microcirculation and lead to the formation of gastric lesions.[7] The disruption in gastric microcirculation can lead to impaired parietal cell function and may result in a reduction of gastric acidity. Although this sequence of events is possible, there are still some discrepancies in the findings on the changes in gastric acidity in response to stress. While some studies have reported an increase in gastric acid secretion,[8,9] others have shown that rats exposed to stress exhibit a reduction in the gastric acidity.[10,11]

Gastrin is a major physiological regulator of gastric acid secretion. It also has an important trophic or growth-promoting influence on the gastric mucosa. Gastrin released from the enteral G cells is one of the main stimuli for acid secretion. Although the increase in gastrin causes an increase in a major aggressive factor, the gastric acid, gastrin also enhances the defense system, such as the gastric microcirculation and the protective mucus, thus creating a balance in the gastric mucosa.[12]

Prostaglandins (PGE) are believed to maintain the integrity of the gastric mucosa by stimulating secretion of mucus and bicarbonate and modulating mucosal blood flow.[13] The effects of PGE_2_ on the gastrointestinal tract (GIT) include inhibition of gastric acid secretion and stimulation of contraction of the longitudinal muscle and relaxation of the circular muscle.[14] Previous studies have shown that after 4 h of water immersion restraint stress (WRS) PGE_2_ levels were decreased by 48% as compared to control and there was significant increase in the formation of lesions.[15,16]

Several studies suggest the involvement of oxidative stress in the etiology of stress-induced gastric lesions.[1,17,18] Pretreatment with vitamin E - tocopherol (TF) and tocotrienol (TT)[1,8,19] - has been demonstrated to prevent gastric mucosal lesion development in rats exposed to stress. Although both possess antioxidant properties,[20] TT was reported to be a better antioxidant than TF.[21]

In this study, we used vitamin E that was extracted from palm oil; this contains approximately 22% TF and 78% TT.[22] The present study aimed to investigate the effects of palm vitamin E on gastric acidity, plasma gastrin level, gastric PGE_2_, and gastric lesions in rats exposed to WRS and to compare the effects of palm vitamin E and α-tocopherol on the parameters measured.

## Materials and Methods

Rats used for this study were kept on a regular night/day cycle, with natural light for a period of 10 h (0700 to 1700 h). Throughout the feeding period all rats were habituated to handling to reduce stress-related disturbances. The rats were housed in large cages with wire-mesh bottoms to prevent coprophagy. Food and water were given *ad libitum* throughout the experiment. This study was approved by the Animal Care and Use Committee of the Faculty of Medicine, Universiti Kebangsaan Malaysia (approval number: FAR/2004/AZLINA/12-JULY/129).

Sixty male Sprague-Dawley rats were divided into three equal groups. The control group was fed with normal rat chow (RC); the treatment groups received the same diet with oral supplements of either palm vitamin E (PVE) or α-tocopherol (α-TF) at 60 mg/kg body weight for 28 days. At the end of treatment period, the 20 rats in each group were further divided into two subgroups: nonstress (NS) and stress groups. Rats in the stress groups were exposed to WRS once at the end of the treatment period. Stress-induced gastric lesions, gastric acid concentration, plasma gastrin level, and gastric PGE_2_ level were measured in all the rats. All measurements were done immediately after the rats were killed at the end of the experiment.

## WRS Model

In WRS, rats were placed in individual plastic restrainers and then immersed in water at 23°C for 3.5 h.[23] Following the restraining procedure, blood was drawn and the rats were killed. Gastric acid was collected and the acid concentration was measured immediately. The stomach was opened along the greater curvature and examined for lesions.

## Determination of Gastric Acidity

Measurement of the gastric acidity was done following a method described by Shay *et al*. (1954). The junctions between the stomach and the esophagus and the duodenum and pylorus were secured before the stomach was isolated. Then 3 ml of distilled water was introduced into the stomach and the organ was carefully shaken. The gastric juice was then collected and centrifuged for 10 min at 3000 rpm. The supernatant was the taken and diluted 10 times. Following this, a few drops of phenolphthalein was added to the solution. Titration was done using 0.01 M solutions until the color of the test solution changed to light pink, indicating pH 7.0. The volume of sodium hydroxide (NaOH) needed for titration was used in the calculation to derive the hydrogen ion concentration.[24]

## Measurement of Plasma Gastrin Level

Blood collected immediately after the exposure to WRS was cooled in an ice bath. Plasma was separated by centrifugation at 3000 rpm at 4°C. Gastrin level was measured using an enzyme immunoassay (EIA) kit (EK-027-04, Phoenix Pharmaceuticals, USA).

## Measurement of Gastric PGE2 Content

Sample preparation for PGE_2_ assay was done following the method described by Redfern *et al*.[25] PGE_2_ was measured using an enzyme immunoassay (EIA) kit (514010, Cayman Chemical, USA).

## Gastric Lesions

The microscopic assessment of the stress-induced gastric lesions in the gastric mucosa was performed by two independent examiners who were blinded to the treatment that the rats received. The assessment of lesions was done according to a quantitative scale. Lesion size in millimeters was determined by measuring each lesion at its greatest diameter. Five petechiae lesion is equal to a 1 mm lesion. The total lengths in each group of rats were averaged and expressed as the lesion index; this method was previously described by Wong *et al*.[26]

### Statistics

Statistical analysis was carried out using the SPSS statistical package, version 12 (SPSS Inc. USA). Normal distribution of all variables was examined by the Kolmogrov-Smirnov test. The results showed that all variables were normally distributed. The results are expressed as means ± SD. Statistical significance (*P* < 0.05) was determined by ANOVA and Tukey's post-hoc test.

## Results

### Effect of WRS on gastric acidity

**Figure 1:**
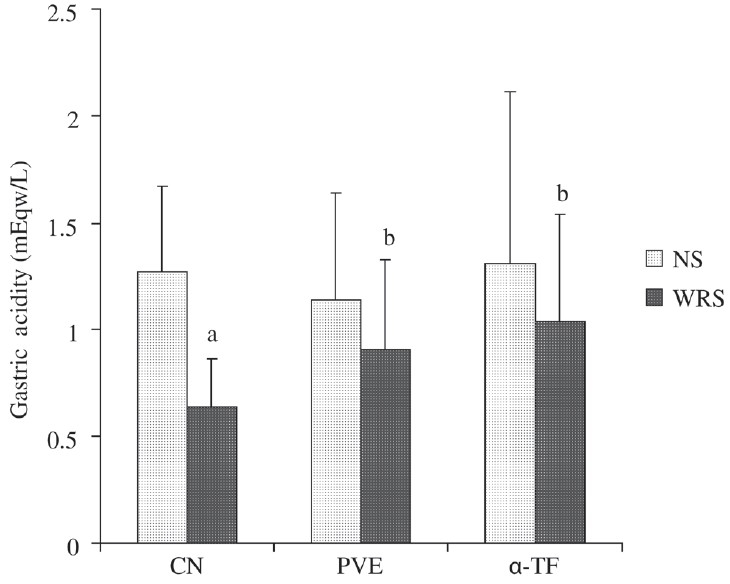
Effects of PVE and α-TF on gastric acidity in rats exposed to water immersion restraint stress (*n* = 10). Each bar represents mean ± SD. a *vs* Nonstress control (NS) (*P* < 0.05); b *vs* Stressed control (*P* < 0.05)

Figure 1 shows the results for gastric acidity. No difference in the gastric acidity between the nonstress groups was observed. Gastric acidity in the control stressed group was reduced by 49%; this reduction was significant (*P* = 0.003) compared to the nonstressed control. Gastric acidity of stressed groups receiving PVE (*P* = 0.049) and α-TF (*P* = 0.046) increased significantly compared to the stressed control. We found no significant difference between the gastric acidity level between the PVE and the α-TF stressed groups.

### Effect of WRS on gastrin level

**Figure 2:**
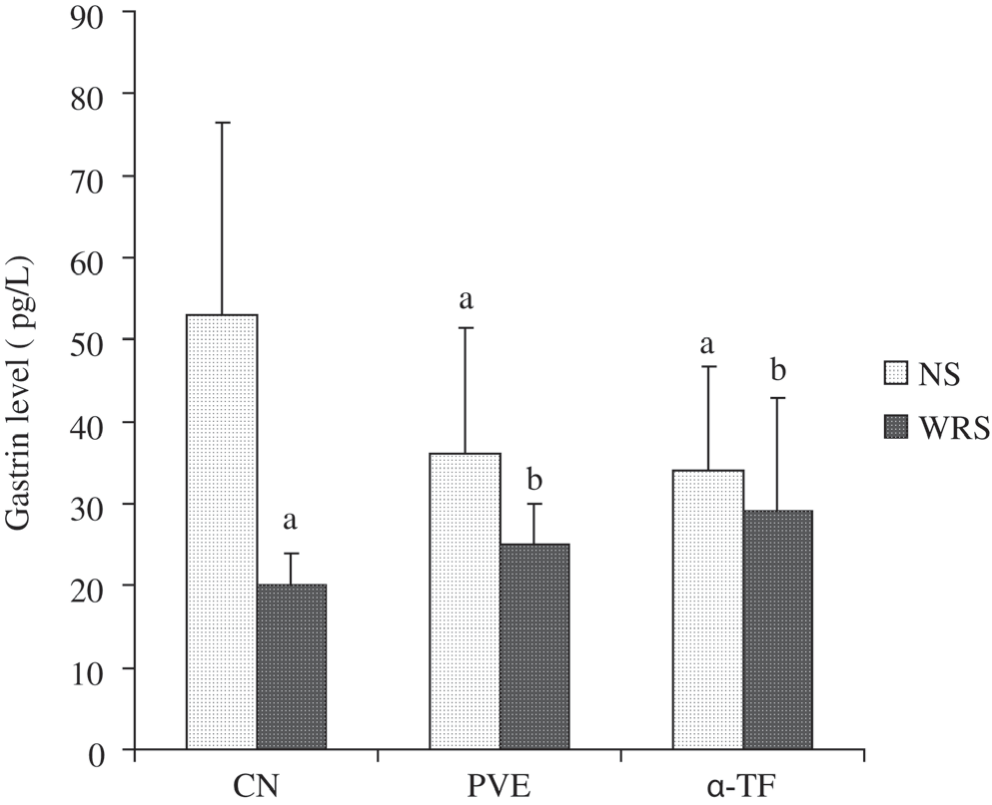
Effects of PVE and α-TF on gastrin level in rats exposed to water immersion restraint stress (*n* = 10). Each bar represents mean ± SD. a vs Nonstress control (NS) (*P* < 0.05); b *vs* Stressed control (*P* < 0.05)

Figure 2 shows that exposure to WRS reduced gastrin level by 62% (*P* = 0.003). The gastrin level of stressed PVE (*P* = 0.033) and α-TF groups (*P* = 0.034) were increased significantly as compared to the stressed control. There was no difference in the gastrin level between nonstressed PVE and α-TF groups. In the nonstressed PVE and α-TF groups, the gastrin levels were reduced significantly in comparison to the nonstressed control.

### Effect of WRS on gastric PGE_2_ content

**Figure 3:**
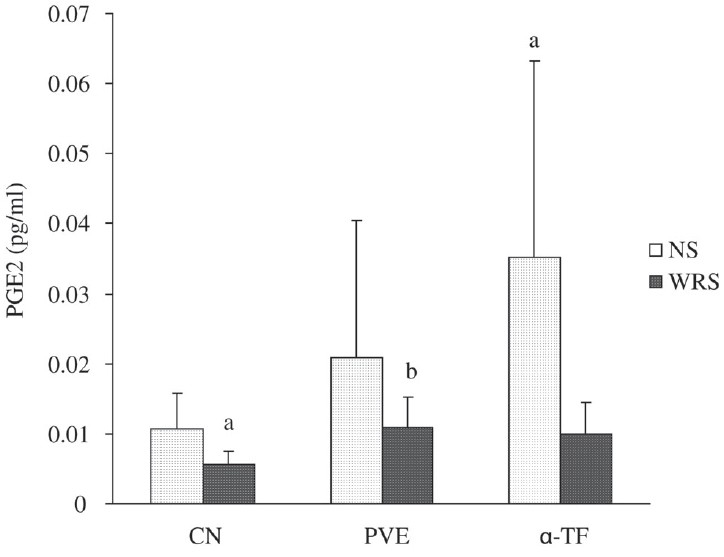
Effects of PVE and α-TF on gastric PGE_2_ level in rats exposed to water immersion restraint stress (*n* = 10). Each bar represents mean ± SD. a *vs* Nonstress control (NS) (*P* < 0.05); b *vs* Stressed control (*P* < 0.05)

Stress reduced the gastric PGE_2_ content significantly compared to nonstress control [Figure 3]. The gastric PGE_2_ content of stressed PVE group (*P* = 0.010) and stressed α-TF group (*P* = 0.043) were increased significantly in comparison to the stressed control. The gastric PGE_2_ content was not different between stressed PVE and stressed α-TF groups. In the nonstressed rats treated with α-TF, the gastric PGE_2_ content was significantly higher than in the nonstressed control group.

### Effect of WRS on gastric lesion

**Figure 4:**
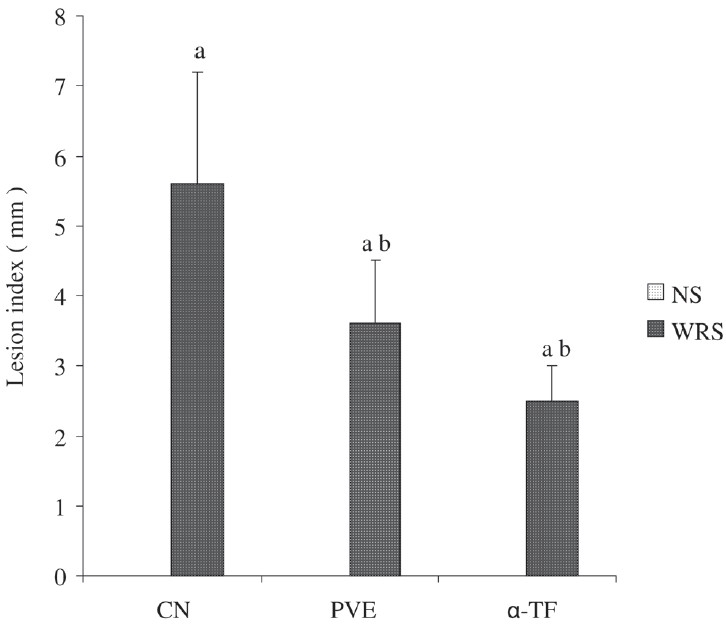
Effects of PVE and α-TF on gastric lesions in rats exposed to water immersion restraint stress (*n* = 10). Each bar represents mean ± SD. a *vs* Nonstress control (NS) (*P* < 0.05); b *vs* Stressed control (*P* < 0.05)

Nonstressed rats showed no focal lesions in the gastric mucosa. However, in the stressed groups there was a significant formation of lesions in stomach. Pretreatments with PVE (*P* = 0.002) and α-TF (*P* = 0.001) reduced gastric lesions significantly in rats exposed to stress. The gastric lesion index was similar in the PVE and the α-TF stressed groups, as shown in Figure 4.

## Discussion

Several studies have been done to prove the protective effect of vitamin E against ulcers or lesion formation in the stomach.[8,19,27] In 1939, Seyle recognized that when activated by stress the physiological system may not only protect or restore but also damage the body. In our study, WRS for 3.5 h reduced gastric acidity significantly. This result is in agreement with the finding of Nur Azlina *et al*.,[19] although their study used a different model of stress, ie, restraint stress alone. Hayase and Takeuchi[10] found that in pylorus-ligated rats, acid secretion decreased in response to restraint alone or to restraint plus water immersion stress for 3.5 h, and there was a significant difference in the gastric juice volume and acid output between the control and restraint plus water immersion groups. In their study, both groups exposed to stress for 7 h exhibited a significant increase in acid secretory activity in terms of the juice volume and the acid output as compared to the controls.[10] This disparity in the results may be due to the longer duration of exposure to WRS that led to increased gastric acidity in rats.

It is also possible that the reduction in the gastric acidity in stress is a result of dysfunction of gastric acid secreting cells. The dysfunction could be due to ischemia of the parietal cells as a result of the compromised gastric microcirculation under stress conditions.[10] This relationship between gastric acidity and gastric blood flow had been shown previously by various researches.[10,28]

In the present study, we found that the gastric acidity of the PVE and α-TF groups were increased significantly after exposure to WRS compared to the control. This finding could be due to the ability of PVE and α-TF to improve gastric acid secretion by minimizing the damage to the gastric acid secreting cells, probably by scavenging the free radicals that are produced during stress.[8]

Our study found that exposure to WRS leads to a reduced gastrin level. The gastrin levels in the stressed PVE group and α-TF group increased significantly compared to the stressed control. Reduction in gastrin levels may not only cause reduction in acid secretion but may also reduce the protective effects of gastrin on the gastric mucosa, which can eventually lead to the formation of lesions in an impaired mucosa.[1]

Gastrin, while increasing gastric acid secretion, at the same time also promotes gastric protection by inducing increased gastric blood flow and causing thickening of gastric mucus.[29] The end effect is the maintenance of the balance between the aggressive and the defensive factors in the gastric mucosa. Administration of a gastrin analog has been reported to protect rats against ethanol-induced gastric injuries.[30,31] Thus, it could be important to preserve gastrin levels in conditions where gastric mucosal damage is likely.

Our present study shows that gastric PGE_2_ content after 3.5 h exposure to WRS was significantly suppressed compared to that of the control group. These findings are consistent with a previous report by Konturek *et al*.[30] and Kato *et al*.[15] Brzozowski *et al*.[32] suggested that the expression of COX-2 mRNA after WRS might be due to deficient PGE_2_ generation in the gastric mucosa. Thus, this expression might reflect the suppression of PGE_2_ generation because COX-2 plays a crucial role in the healing of gastric ulcers.[15]

Brzozowski *et al*.[33] found that exposure to stress led to ischemia-reperfusion which produced a significant fall in PGE_2_ generation in the gastric mucosa; however, PGE_2_ was gradually restored during mucosal recovery from gastric lesions, suggesting that endogenous prostaglandin may be involved in the spontaneous healing of these lesions. This is supported by the fact that PGE_2_ generation reached higher values in ulcerated gastric mucosa during the course of healing than it did in nonulcerated mucosa.

Our results showed that the gastric PGE_2_ content of nonstressed α-TF group increased significantly. The gastric PGE_2_ content of stressed PVE and stressed α-TF groups increased significantly compared to the stressed control. The improvement of the gastric PGE_2_ content could possibly be due to the effect of vitamin E, which stimulates prostaglandin synthesis by activating the calcium-dependent phospholipase enzyme A2 and by inhibiting the lipooxygenase enzyme.[34] Thus, pretreatment of animals with vitamin E may prevent the gastric mucosal damage caused by stress by increasing PGE_2_ level.

Azlina *et al*.[28] found that the gastric PGE_2_ content in all the groups studied were not significantly different, despite the reduced PGE_2_ content after exposure to stress. However, in groups supplemented with TT or TF, the gastric PGE_2_ content was increased. Their finding suggested that stress did not alter the gastric PGE_2_ content and supplementation with TT or TF did not change the gastric PGE content in stressed rats. The difference in their findings compared to ours could be due to the difference in the models of inducing stress, which suggests that different stress models can induce the formation of gastric lesions through different pathologic mechanisms.

Prostaglandins, one of the major groups of chemical mediators in the mammalian body, are involved in numerous physiological reactions, such as inflammation and cellular differentiation.[35] Prostaglandins, especially PGE_2_, also have cytoprotective effects on gastric mucosa as a consequence of various physiological mechanisms, including increased epithelial mucus and bicarbonate secretion,[36] amelioration of mucosal blood flow,[37] and inhibition of free radical activities and enzyme release from neutrophils.[38]

In the present study, gastric lesions developed in the gastric mucosa in response to restraint plus water immersion stress for 3.5 h. This result is in agreement with the findings of Konturek *et al*.;[30] they suggested that the increased formation of the gastric lesions might be due to the increased gastric contractions, which resulted in temporary restriction of blood flow to the mucosa and produced anoxic damage. The restriction of blood flow might also involve vascular contraction or the shunting of blood away from the mucosa, thereby accentuating anoxic damage.[39]

Our data showed that in rats exposed to WRS, the reduction in gastric PGE_2_ content was negatively correlated with the increase in lesion formation. This correlation explains the role of PGE_2_ in protecting the gastric mucosa against the formation of lesions. It proved the ability of vitamin E to improve the level of gastric PGE_2_ content, which has an important role in protecting the gastric mucosa.

We conclude that exposure to WRS causes decrease in gastric acidity, gastrin level, and gastric PGE_2_ content and increase in the formation of gastric lesions. Treatment with PVE or α-TF can prevent gastric mucosal damage caused by stress by increasing PGE_2_ level and blocking the changes in gastric acidity and gastrin level. We postulate that the protective effects of both PVE and α-TF could be due to their ability to preserve the gastrin levels and the gastric PGE_2_ level in conditions of stress, which results in the maintenance of the trophic action of these hormones in the gastric mucosa, a desirable factor in the preservation of gastric mucosal integrity.
